# Transgenerational programming of maternal behaviour by prenatal stress

**DOI:** 10.1186/1471-2393-13-S1-S9

**Published:** 2013-01-31

**Authors:** Isaac D Ward, Fabíola C R Zucchi, Jerrah C Robbins, Erin A Falkenberg, David M Olson, Karen Benzies, Gerlinde A Metz

**Affiliations:** 1 Canadian Centre for Behavioural Neuroscience, University of Lethbridge, 4401 University Drive, Lethbridge, AB, Canada T1K3M4; 2 Departments of Obstetrics & Gynecology, Pediatrics and Physiology, University of Alberta, 227 HMRC, University of Alberta, Edmonton, AB, Canada T6G2S2; 3 Faculty of Nursing, University of Calgary, 2500 University Dr. NW, Calgary, AB, Canada T2N1N4

## Abstract

Peripartum events hold the potential to have dramatic effects in the programming of physiology and behaviour of offspring and possibly subsequent generations. Here we have characterized transgenerational changes in rat maternal behaviour as a function of gestational and prenatal stress. Pregnant dams of the parental generation were exposed to stress from days 12-18 (F0-S). Their daughters and grand-daughters were either stressed (F1-SS, F2-SSS) or non-stressed (F1-SN, F2-SNN). Maternal antepartum behaviours were analyzed at a time when pregnant dams usually show a high frequency of tail chasing behaviours. F1-SS, F2-SNN and F2-SSS groups showed a significant reduction in tail chasing behaviours when compared with controls. The effects of multigenerational stress (SSS) slightly exceeded those of transgenerational stress (SNN) and resulted in absence of tail chasing behaviour. These findings suggest that antepartum maternal behaviour in rats is programmed by transgenerational inheritance of stress responses. Thus, altered antepartum maternal behaviour may serve as an indicator of an activated stress response during gestation.

## Introduction

The perinatal period is a time of high vulnerability to environmental influences. It is well established that maternal stress [1] and the quality of maternal care [2,3] influence offspring development and stress responses with consequences potentially lasting to adulthood [4-6]. Notably, maternal care is affected by stress during early post-natal development [7] as well as by stress during gestation [8].

Both stress and maternal care have been reported to program physiology and behaviour across generations [4,9]. Transgenerational programming of stress responses and associated trait anxiety were suggested to transmit to subsequent generations in the absence of stress via germ line-dependent mechanisms [10]. Non-genomic transmission of behavioural traits was also shown for maternal care in Long-Evans rats. Maternal care of offspring was highly correlated to the behaviour exhibited by their own mothers in the first week after parturition [4,11,12]. It was suggested that maternal stress and the mother’s care determine offspring behavioural traits and their stress responses through epigenetic mechanisms [4,10,13-15]. The epigenetic imprinting of adult physiology and behaviour by stress and maternal care suggests that this reciprocal relationship represents a potential target for prevention and intervention to improve offspring health outcomes.

The purpose of this study was to characterize changes in maternal behaviour as a function of transgenerational stress in rats. Antepartum maternal behaviour may offer predictive value as an indicator of an activated stress response and postpartum maternal care. Here we identified changes in maternal tail chasing as a sensitive measure of stress in antepartum maternal behaviour. Ancestral experience may determine the quality of maternal tail chasing and physiological responses to stress in the progeny. We hypothesized that antepartum maternal behaviour is programmed by transgenerational inheritance of stress response and associated with characteristic behavioural change. We compare the effects of gestational stress in the parental generation with the effects of programming by prenatal stress in subsequent generations. Our findings indicate that a reduction in tail chasing behaviour in pregnant dams prior to parturition is reflective of transgenerational programming by stress.

## Methods

### Animals

Twenty-nine female adult Long-Evans Hooded rats, raised at the University of Lethbridge vivarium, were used. Eighteen non-stressed young adult males were used for breeding. During the test period, the pregnant dams were housed individually in standard polycarbonate shoebox cages (45.5 X 25.5 X 20 cm) on corn cob bedding (Bed O Cobs 1 / 8``; The Andersons Lab Bedding, Ohio, USA). Maternal weight gain during pregnancy and litter size were monitored. The light cycle was 12:12 h with lights on at 07:30 h. The housing room was maintained at a temperature of 20°C and 30% relative humidity. The experiments were approved by the University of Lethbridge Animal Welfare Committee (protocol #0803) according to guidelines set forth by the Canadian Council of Animals Care.

### Experimental design

**Figure 1:**
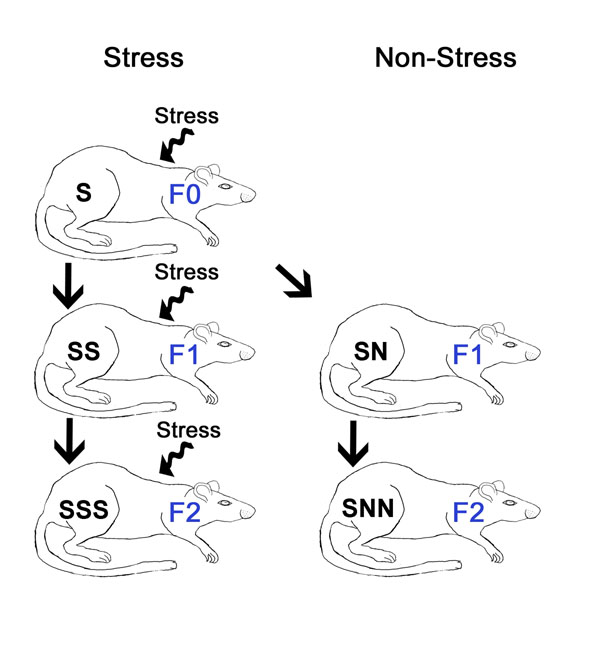
Experimental design to induce prenatal, transgenerational and multigenerational stress. Animals of the parental generation (F0) were assigned to a stress (S) condition. Their pregnant prenatally stressed daughters received either stress (SS) or no stress (SN). Their pregnant grand-daughters received either stress (SSS) or no stress (SNN). Groups of non-stressed controls were bred alongside the stressed generations. In our conceptual framework prenatal stress programs the germ line of F1 progeny, resulting in physiological and behavioural changes in the F2 generation. S, stress; N, non-stress generation.

Three generations of rats were bred (see Figure 1) and subdivided into five groups: (1) Non-stressed controls (n=7); (2) Parental generation stressed during gestation (F0-S; n=6); (3) Prenatally stressed first filial generation that underwent gestational stress (F1-SS; n=5); (4) Second filial generation that originated from a stressed parental generation, but neither they or their mothers were stressed (F2-SNN; n=5); (5) Second gestationally stressed filial generation that received prenatal stress through two previous generations (F2-SSS; n=6). Thus, SNN rats represent a transgenerational condition of stress, while SSS rats represent a multigenerational condition of stress.

### Stress treatment

Gestational/prenatal stress was applied daily from gestational days 12 through 18. Restraint and swim stress were applied daily in a semi-random sequence at 8:30 am and 4:30 pm. This stress regimen corresponds to a mild to moderate stressor in rats [16]. Stress treatments were performed in a designated room other than the housing facility.

#### Restraint

Rats were placed in a transparent Plexiglas container (5 cm inner diameter) for a period of 20 minutes each day [16]. The container had perforated ends to allow ventilation. The container maintained the animals in a standing position without compression of the body.

#### Swim stress

Rats were individually placed in a tub filled with water at room temperature water (~22 degrees Celsius) for five minutes. The water was deep enough so that neither the rat’s feet nor its tail had contact with the bottom. After the 5 minutes dams were towel dried and returned to their home cage.

### Antepartum behavioural analysis

Behaviour was videorecorded using an infrared video surveillance system (Panasonic WV-BP330, Panasonic, Japan). Animals were monitored by the surveillance system starting at 48 hours prior to expected delivery. Behavioural observations were performed for 60-minute intervals from video recorded data. The scoring intervals included the periods of 14-15, 18-19, and 22-23 hours prior to parturition of the first pup. These intervals were chosen because they reveal the most significant changes in maternal behaviour among control rats. The amount of time spent engaged in tail chasing, and the total number of rotations performed were measured.

**Figure 2:**
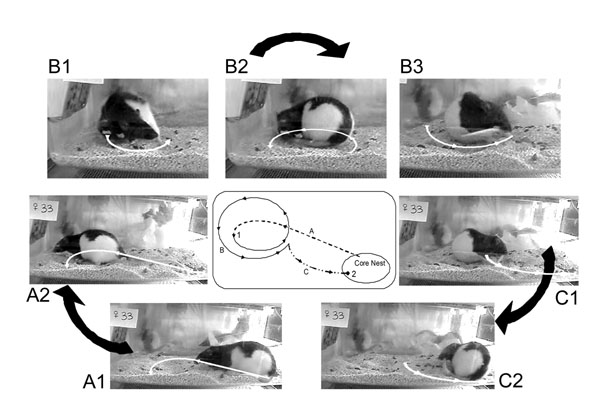
Illustration of a representative sequence of movements comprising tail chasing in a naïve pregnant dam. Photographs in A represent departure from the core nest area. Photographs in B represent engagement with the tail. Photographs in C represent a return to the core nest area. A1) Dam proceeds from core nest, A2) to the open space of her home cage. B1) Dam takes interest in her tail, B2) and rotates in the attempt to catch her tail with the mouth, B3) continuing the rotation while holding the tail in her mouth. C1) Dam with tail in mouth begins return to core nest, C2) and delivers the tail to the core nest. The central map shows a representative trajectory of a horizontal movement sequence in the home cage. Numbers 1 and 2 indicate start and end points of the movement, respectively.

Initiation of tail chasing behaviour was scored when the dam took interest in her tail followed by chasing or holding the tail with the mouth. Completion of a tail chasing event was scored once the rat disengaged with her tail and initiated a different activity. Figure 2A-C illustrates a characteristic sequence of tail chasing behaviour. Tail grooming behaviour was not included in this analysis.

### Statistical analysis

Statistical analysis was performed using a Statview software package (Abacus Concepts, CA, USA). All data were subjected to square root transformation to assure constant variance and Gaussian distribution prior to statistical testing. The data were subject to analysis of variance (ANOVA) for measures across all testing groups followed by post-hoc Fisher’s PLSD test for differences between groups. Paired comparisons between time intervals were performed using paired *t*-tests. Correlation analysis was performed using a Fisher’s r to z transformation. A *p-*value of less than 0.05 was chosen as the significance level for all statistical analyses. All data are presented as the mean ± standard error (S.E.M.).

## Results

### Qualitative analysis: prenatal and transgenerational stress disrupt the patterns of tail chasing behaviour

The effect of prenatal and transgenerational stress on pregnant dams was noticeable from an observational perspective. Non-stress control animals were found to rotate within a specific sequence of events that comprise a departure (Figure 2A), engagement (Figure 2B) and return component (Figure 2C). Typically, a tail chasing event was initiated when a pregnant dam departed from her core nest area and began to engage with her tail (Figure 2A). While engaged with her tail, a dam would usually chase the tail, eventually pick it up and carry it in her mouth (Figure 2B). The dam’s head movement towards the tail usually initiates ipsiversive horizontal rotational movements of the body that are accompanied by coordinated fore- and hind limb steps. Once the dam caught the middle portion or tip of her tail she would carry the tail to her nest with the mouth and drop it at the core nest area (Figure 2C). Prenatal and transgenerational stress disrupted this characteristic sequence of events in that the dam showed reduced interest in her tail and frequently failed to show the departure, engagement and return components of this behaviour.

### Quantitative analysis: prenatal and transgenerational stress reduces the frequency of tail chasing behaviour

Antepartum tail chasing was assessed for time spent tail chasing and total number of rotations prior to parturition.

**Figure 3:**
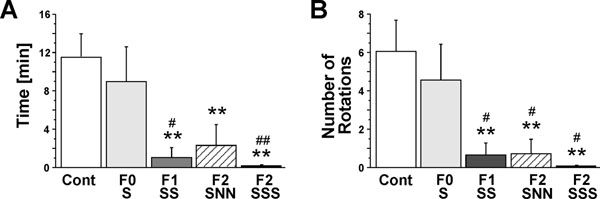
Tail chasing behaviour in parental and transgenerationally stressed rats from 14-15 hours prior to parturition. Prenatal and transgenerational stress reduced the amount of time spent tail chasing (A) and the number of rotations (B) in a 60-min time interval. There was no effect of gestational stress in the parental generation (n=6), however, both transgenerational (F2-SNN, n=5) and multigenerational (F1-SS [n=5], F2-SSS [n=6]) stress reduced tail chasing activity. Cont includes non-stress control dams (n=7). Asterisks indicate statistical significances: ** p≤0.01, compared to control group. ^#^ p≤0.05, ^##^ p≤0.01, compared to parental F0-S generation. Data represent mean square root±SEM.

As displayed in Figure 3A the time spent in tail chasing showed a Group effect (F(4,24)=5.44, p≤0.01, power of 0.94). There was no effect of gestational stress in the parental (F0) generation (p=0.43). F1-SS prenatally and gestationally stressed dams spent significantly less time engaged in tail chasing behaviour (p≤0.01). Furthermore, the F2-SNN animals showed a significant reduction in the time spent in tail chasing behaviours when compared to controls (p≤0.01). The greatest effect was exhibited by the F2-SSS group, in which rotation was absent (p≤0.01), suggesting a cumulative effect of multigenerational stress. Also, there also was a significant reduction in F1-SS and F2-SSS when compared to parental F0-S rats (p≤0.05 and p≤0.01, respectively).

Furthermore, the number of rotations also showed a significant effect of Group (F(4,24)=5.27, p≤0.01, power of 0.91). There was no difference between stressed and non-stressed F0 dams (p=0.38). F1-SS animals performed fewer rotations than control animals (p≤0.01; Figure 3B). In addition, F2-SNN dams (p≤0.01) and F2-SSS dams (p≤0.01) performed fewer rotations than control dams. There was a significant effect of generation because F1-SS, F2-SNN and F2-SSS rotated less than parental F0-S rats (all p’s≤0.05).

Correlation analysis revealed that neither time spent tail chasing (weight gain: r=0.077; litter size: r=0.173) nor the number of rotations (weight gain: r=0.071; litter size: r=0.145) were related to maternal weight gain or litter size.

**Figure 4:**
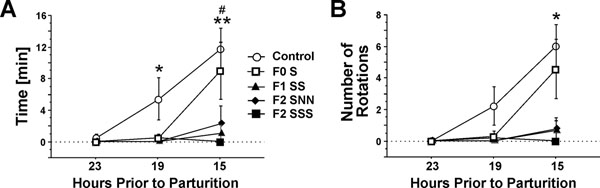
Time course of time spent tail chasing (A) and the number of rotations performed (B) in parental and transgenerationally stressed rats at 23, 19, and 15 hours prior to parturition. Tail chasing in control (n=7) and parental F0-S (n=6) dams increased from 23 to 15 hours prior to parturition. Note that there was no increase in tail chasing behaviours in transgenerational (F2-SNN, n=5) and multigenerational (F1-SS [n=5], F2-SSS [n=6]) stressed animals. Asterisks indicate statistical significances: * p≤0.05 and ** p≤0.01 in control dams, ^#^ p≤0.05 in F0-S dams, compared to the 23-hour time interval. Data represent mean square root±SEM.

The time course of changes from interval 23, 19 and 15 hours revealed a main effect of Group (F(4,24)=5.82, p≤0.01; F(2,24)=4.86, p≤0.01), Interval (F(2,24)=14.48, p ≤0.001; F(2,24)=14.77, p≤0.001) and a Group by Time interaction (F(8,48)=3.75, p≤0.01; F(8,48)=4.19, p≤0.001) for the time spent tail chasing and the number of rotations, respectively. Control dams spent significantly more time chasing their tail at 19 hours (t=2.98, p≤0.05) and 15 hours (t=4.49, p≤0.01) prior to parturition compared to the 23-hour time point (Figure 4A). They also performed more rotations at 15 hours than at 23 hours prior to parturition (t=2.91, p≤0.05; Figure 4B). The time of F0-S dams increased from 19 to 15 hours (t=2.57, p≤0.05). There was no change in tail chasing behaviour in F2 or F3 animals.

## Discussion

The purpose of this study was to determine behavioural changes that occur prior to parturition as a result of gestational, prenatal and transgenerational stress. Using tail chasing as a new measure of antepartum maternal behaviour, we show that prenatal and transgenerational stress, but not gestational stress, alters this behaviour. We observed a significant decrease in the amount of tail chasing over that of controls during the antepartum period in F1 and F2 mothers that had experienced prenatal stress. We show that the effects of prenatal stress as expressed by reduced tail chasing are passed on to the next generation and its progeny. The effects of prenatal stress persist in the absence of stress in the filial generations, suggesting physiological and behavioural programming in the offspring with possibly lifelong consequences.

To date there has been very little investigation of rodent antepartum behaviours. We propose that maternal behaviour during the antepartum period may reflect preparatory activities, such as nest building. A previous study showed that nest building activities of the dam undergoes a significant increase during the 24 hours prior to parturition [17], which is in agreement with the time course of tail chasing behaviour. In the present study, however, dams were not provided with nest building material for better visibility during video analysis and thus no unequivocal correlations between tail chasing and nest building activities are possible. It is possible that the antepartum increase in tail chasing behaviour is indicative of post-partum maternal care. Maternal care, including licking and grooming as a form of tactile stimulation, has been shown to reduce the behavioural and endocrine consequences of preterm birth or early environmental adversity in rodents and human infants [18-21]. Tail chasing may also be indicative of other forms of maternal care, such as the retrieval of pups. The observation that the pregnant dam typically engages with the tail outside of her core nest area and completes the tail chasing bout by carrying it back to her nest would supports this hypothesis. Although the specific function of maternal tail chasing behaviour remains to be determined, the present findings suggest that antepartum maternal behaviour may represent a valid indicator of post-partum maternal care.

Periparturitional maternal behaviours may be particularly sensitive to the effects of stress. Prenatal stress may permanently alter brain development, which may manifest in altered nest building behaviour and behavioural simplification when a prenatally stressed rat matures and becomes pregnant [22]. Furthermore, corticosterone levels in pregnant rats peak on gestational day 18 and remain high until parturition [23]. The intricate endocrine changes of gestation and the rise in antepartum corticosterone levels in particular may stimulate central dopaminergic systems and lead to greater locomotor activity [24]. Thus, greater engagement in tail chasing behaviour in dams may be causally related to enhanced hypothalamo-pituitary-adrenal (HPA) axis activity preparing for parturition. It is possible, however, that HPA axis programming by prenatal and transgenerational stress reduces overall motor activity and leads to reduced tail chasing behaviour [25]. Furthermore, prenatal stress may alter basal activity of the HPA axis and the response to stress in adulthood [1,26]. The resulting imbalance of glucocorticoid-regulated endocrine factors participating in parturition may contribute to altering antepartum maternal behaviours.

Altered maternal behaviour during gestation may also reflect changes in profiles of progesterone levels in rodents. In rats, parturition is associated with a decrease in progesterone production, also termed progesterone withdrawal [27]. Progesterone plasma levels usually begin to decline on gestational day 19 [28]. In the present study, tail chasing behaviour was analyzed on gestational day 22, 23, 19 and 15 hours prior to parturition, at a time of low progesterone levels. The time course of tail chasing behaviour in the 24 hours leading to parturition suggest that endocrine changes may mediate an increase or decrease in this activity. While gestational stress in the parental generation did not affect tail chasing behaviour and likely did not affect progesterone levels, prenatal and transgenerational stress may have diminished the engagement in tail chasing through interference with progesterone regulation and other components. This notion is supported by a study showing that the onset of maternal nest building at or about the end of pregnancy is associated with a fall in circulating levels of progesterone in rabbits [29]. Furthermore, inadequacy or absence of a nest area can adversely affect maternal care [29]. Notably, gestational stress can dysregulate progesterone formation in juvenile offspring [30], a change that may persist into adulthood in female F1 and F2 animals to perturb physiological and behavioural adjustments to pregnancy. If any of these changes contributed to the present behavioural observations, our data show that the underlying endocrine processes were not associated with profound maternal weight or litter size effects.

The influence of transgenerational programming by prenatal stress was evident in the F2-SNN generation. Although an F3 generation would be necessary to confirm truly epigenetic effects [31,32], our findings suggest that programming by prenatal stress disrupts tail chasing behaviour in the grand-offspring and great-grand-offspring. These effects may be due to direct germ line exposure to maternal stress in the womb. Moreover, the exposure to multi-generational stress in F1-SS and F2-SSS rats indicates that prenatal stress has cumulative, context-dependent consequences. These findings suggest that epigenetic mechanisms may mediate a gradually altering physiological response to recurrent stress in each generation. The formation of an epigenetic memory to a single or recurrent adverse event within a family history may assist in adjusting physiological and/or behavioural patterns to a stressful environment. Epigenetic memory refers to transgenerationally stable, yet dynamic re-programming of the germline epigenome that transfers information across generations in the absence of changes in DNA sequence [33,34]. Through this kind of memory, the trait of altered maternal behaviour may be passed on to the subsequent generation [13,35,36] via, for example, a heritable pattern of hypermethylation of the gene encoding brain-derived neurotrophic factor (BDNF) [37]. In the offspring, the resulting reduction in BDNF expression and low levels of this growth factor in the prefrontal cortex during development may have drastic consequences for cognitive and affective abilities and the response to stress in adulthood.

In conclusion, our findings show that antepartum maternal behaviour may have particularly predictive value of an activated stress response, parturition and post-partum maternal care towards her offspring. Importantly, prenatal stress may program physiological and behavioural responses to pregnancy and postpartum maternal care in subsequent generations and their progeny.

## Author contributions

IDW, FCRZ, DMO, KB and GAM designed the study. IDW, FCRZ, JCR, and EAF performed the experiments, collected and analyzed the data. IDW and GAM completed the statistical analysis and prepared the figures. IDW, FCRZ, KB and GAM wrote the manuscript.

## Competing interests

The authors declare no competing financial interests.

## Declarations

This article has been published as part of *BMC Pregnancy and Childbirth* Volume 13 Supplement 1, 2013: Preterm Birth: Interdisciplinary Research from the Preterm Birth and Healthy Outcomes Team (PreHOT). The full contents of the supplement are available online at http://www.biomedcentral.com/bmcpregnancychildbirth/supplements/13/S1.

All of the publication fees will be funded by the Preterm Birth and Healthy Outcomes Team Interdisciplinary Team Grant (#200700595) from Alberta Innovates - Health Solutions, formerly the Alberta Heritage Foundation for Medical Research.
